# Recurrent risk of ischemic stroke due to Vertebrobasilar Dolichoectasia

**DOI:** 10.1186/s12883-019-1400-9

**Published:** 2019-07-17

**Authors:** Zhaoyao Chen, Shuai Zhang, Zhengze Dai, Xi Cheng, Minghua Wu, Qiliang Dai, Xinfeng Liu, Gelin Xu

**Affiliations:** 1 Department of Neurology, Jinling Clinical College of Nanjing Medical University, Nanjing, 210002 Jiangsu China; 2 Department of Neurology, Jiangsu Province Hospital of Chinese Medicine, Affiliated Hospital of Nanjing University of Chinese Medicine, Nanjing, 210002 Jiangsu China; 3 Department of Neurology, The Affiliated Hospital of Yangzhou University, Yangzhou University, Yangzhou, 211400 Jiangsu China; 4 Department of Neurology, Nanjing Pukou Hospital, Nanjing, 210002 Jiangsu China; 5 Department of Geriatrics, the First Affiliation Hospital of Nanjing Medical University, Nanjing, 210002 Jiangsu China; 6 Department of Neurology, Jinling Hospital, Medical College of Nanjing University, 305# East Zhongshan Road, Nanjing, 210002 Jiangsu China

**Keywords:** Vertebrobasilar Dolichoectasia, Stroke recurrence, Intracranial atherosclerosis, Ischemic heart disease

## Abstract

**Background:**

Patients with vertebrobasilar dolichoectasia usually have persistent hemodynamic abnormalities, and therefore, may bear an increased risk of stroke. This study aimed to identify risk factors for stroke recurrence in patients with vertebrobasilar dolichoectasia.

**Methods:**

Patients with acute ischemic stroke were screened and evaluated for eligibility. Enrolled patients were followed via scheduled clinical visits or telephone interviews. Ischemic stroke recurrence was proposed with clinical symptoms and confirmed with cranial Magnetic Resonance Imaging or Computerized Tomography scans. Baseline characteristics and vascular geometry were compared between patients with and without stroke recurrence. Significant parameters were introduced into COX proportional hazard model to detect possible predictors of stroke recurrence.

**Results:**

A total of 115 stroke patients with vertebrobasilar dolichoectasia were enrolled, of which 22 (19.1%) had recurrence during 22 ± 6 months follow-up. Basilar artery diameter ≥ 5.3 mm (HR = 4.744; 95% CI, 1.718–13.097; *P* = 0.003), diffuse intracranial dolichoectasia (HR = 3.603; 95% CI, 1.367–9.496; *P* = 0.010) and ischemic heart disease history (HR = 4.095; 95% CI, 1.221–13.740; *P =* 0.022) had increased risk of recurrence.

**Conclusions:**

Stroke patients with vertebrobasilar dolichoectasia may have a high risk of recurrence. Larger basilar artery diameter or diffuse intracranial dolichoectasia may increase the risk of recurrence.

## Background

Vertebrobasilar dolichoectasia (VBD) has been associated with stroke and all-cause mortality [1]. Subsequent stroke risk is also higher in patients with VBD, with ischemic stroke (IS) and transient ischemic attack (TIA) being the most common types of recurrence [2, 3]. In addition to common risk factors such as hypertension, obesity, diabetes mellitus, smoking [4], and ethnicity [5], dolichoectasia of intracranial arteries, which can subsequently lead to intraluminal thrombosis, or perforator vessel occlusion through stretching or squeezing, may also increase the risk of stroke recurrence [2].

Previous studies on stroke recurrence in patients with VBD either had relatively small sample sizes [3, 6], did not include patients with dolichoectasia of anterior circulation [2] or lacked evaluation of total arterial geometry abnormalities. Also, studies of VBD in the Han Chinese population are limited. In this study, we included baseline characters of the patients and in depth descriptions of the arterial geometry, such as dolichoectasia features and arterial stenosis, to help identify the predictive factors for IS recurrence in VBD patients.

## Methods

For this stroke-registry based retrospective cohort study, we retrieved data from Nanjing Stroke Registry Program [7] and The Affiliated Hospital of Nanjing University of Chinese Medicine. The study was approved by the local research ethics committees of Jinling Hospital and Jiangsu Province Hospital of Chinese Medicine and conducted in accordance with the Declaration of Helsinki. All participants or their guardians gave written informed consent.

### Participants

**Fig. 1:**
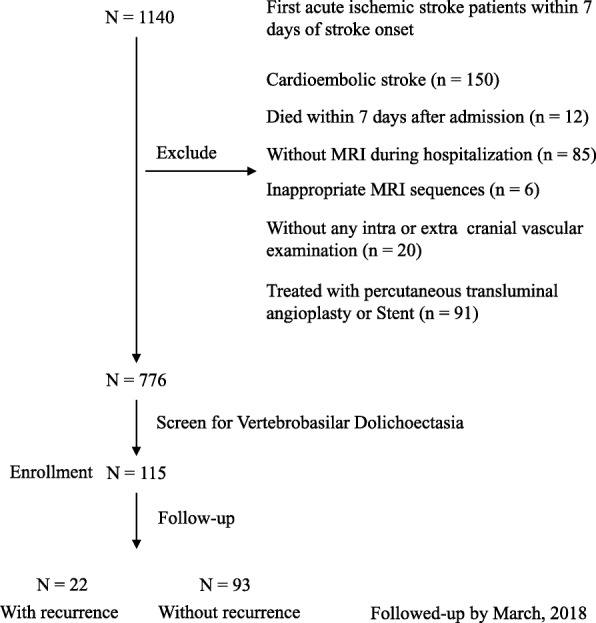
Flow-Chart of Patient Enrollment in This Study

Consecutive patients with first acute IS within 7 days of stroke onset from September 1, 2015 to August 31, 2016 were included in this study. All ischemic stroke events were confirmed by magnetic resonance imaging (MRI) or computerized tomography (CT). We excluded patients with brain hemorrhage, subarachnoid hemorrhage, traumatic brain injury, venous sinus thrombosis, cardioembolic stroke, or treated with percutaneous transluminal angioplasty and stent; patients who died within 7 days were also excluded because their diagnosis of stroke recurrence was mostly undetermined. Besides, patients were excluded if they had no brain MRI/MRA or CT/CTA or digital subtraction angiography (DSA) which would be needed for the measurement of intracranial arterial diameter and determining if intracranial arterial dolichoectasia (IADE) or intracranial atherosclerosis (ICAS) is present. A flowchart of patient enrollment is shown in Fig. 1.

### Diagnosis of VBD and ICAS

The diameter of main seven intracranial arteries, include the basilar artery (BA) at mid-pons, the intracranial vertebral arteries (VA) (R and L) at the V4 segment, the internal carotid arteries (ICA) (R and L) at the intracavernous segment and the middle cerebral arteries (MCA) (R and L) at the M1 segment, were measured. We focused on the basilar artery in this study because it is the most commonly affected dolichoectasia artery [8]. VBD is defined according to widely accepted criteria [9, 10]. Ectasia was diagnosed if the BA diameter ≥ 4.6 mm and elongated or enlarged over its entire course; not including segmental saccular enlargement or giant aneurysms (diameter > 25 mm) which were described as translational type [11]. A semi-quantitative four-point scale was used to determine the elongation of BA: the height of BA bifurcation was scored as 0 (at or below the dorsum sellae), 1 (within the suprasellar cistern), 2 (at the third ventricle floor) and 3 (indentation and elevation of the third ventricle floor). The laterality of BA was scored as 0 (midline throughout), 1 (R or L, the medial-to-lateral margin of clivus or dorsum sellae), 2 (R or L, the lateral-to-lateral margin of clivus or dorsum sellae) and 3 (R or L, at cerebellopontine angle cistern). Dolichos was diagnosed if the height of BA bifurcation or laterality of BA ≥2 scale. Diagnosed criteria for anterior circulation dolichoectasia is limited; we defined this condition if ICA or MCA was circuitous or enlargement on visual impression and an ICA diameter ≥ 7 mm or a MCA diameter ≥ 4 mm according to a previous study [1]. Diffuse intracranial dolichoectasia was defined with dilation of at less two intracranial arterial supply system (for example, vertebrobasilar system, left or right anterior circulation) [12]. Intra-rater and inter-rater reliability for determinate the existing of VBD or not were 0.821 and 0.796, respectively.

Intracranial arterial stenoses on ICA distal to the ophthalmic artery, anterior cerebral artery (ACA) A1/A2, MCA M1/M2, posterior cerebral artery (PCA) P1/P2, BA, and intracranial VA were measured on 3D-TOF MRA, CTA, or DSA. Stenoses were grouped as < 50, 50 to 69%, 70 to 99%, and occlusion according to previous established criteria [13]. The extracranial arterial stenosis was examined by CTA, contrast-enhanced MRA, or DSA [14]. If a patient experienced with more than one radiological spectrum, the degree of cerebral artery stenosis calculated on DSA image was finally adopted. We defined the presence of ICAS or extracranial atherosclerosis (ECAS) once a cerebral artery has ≥50% stenosis or occlusion.

### Baseline assessments

Baseline demographics, vascular risk factors, in hospital treatment, and medical records were retrieved. Vascular risk factors including hypertension, diabetes mellitus, hyperlipidemia, ischemic heart disease and smoking was recorded. All patients had brain MRI (sequences included axial T1-weighted, axial T2-weighted, axial diffusion-weighted imaging, fluid-attenuated inversion recovery, and in most cases, 3D time-of-flight MRA) in either a 3.0-T (MAGENTOM Trio, Siemens, Erlangen, Germany) or a 1.5-T (GE Medical Systems, Milwaukee, WI) system. The image data was stored in Digital Imaging Communications in Medicine (DICOM) format for further analysis in Centricity Enterprise Web 3.0 Client (GE Medical Systems, Milwaukee, WI). All MRI source and maximum intensity projection (MIP) images were evaluated by two neurologists who were blinded to clinical information.

### Follow-up assessments

Patients were offered either a face-to-face interview or a telephone interview every 3 months by trained research doctors. The guardians were contacted if patients were died or can’t help themselves with the inquiry. The follow-up interval was defined as the time between the stroke onset and contact or decease. The primary endpoint was IS recurrence. Each recurrence was verified by a stroke specialist’s review of the medical records, including confirmation by CT or MRI, especially to rule out any stroke mimics [15].

### Statistical analysis

Continuous variables were recorded as mean ± SD or as median [IQR]. Categorical variables were presented as proportions. Between-group comparisons of the distribution of continuous variables were performed using the independent samples t-test or Mann–Whitney U test. Comparisons of categorical variables were performed using the χ^2^ test or Fisher-exact test. Risk factors with a *P* value of < 0.1 in the univariate analysis were included in multivariate Cox proportional hazards regression forward: LR model, to analyze levels of hazard ratio (HR) of recurrence. All statistical testing was two-tailed; *P* < 0.05 was considered statistically significant. All analyses were performed in PASW Statistics 18.0 (SPSS Inc., Chicago, IL).

## Results

One hundred and-fifteen VBD patients were consecutively included with a mean age of 63.1 ± 10.7 years (ranged from 41 to 83 years); 88 (76.5%) patients were men. Other baseline demographic and clinical characteristics are delineated in Table 1; 85 (73.9%) patients had hypertension, 38 (33.0%) had diabetes mellitus, and 10 (8.7%) patients had a history of ischemic heart disease (IHD). In addition, 34 (29.6%) had hyperlipidemia and 37 (32.2%) smoked. The median National Institute of Health stroke scale (NIHSS) score at admission was 3 (IQR 1–6). The general treatment during hospitalization included in this study referred to antiplatelet (85.5%) or anticoagulant (20.0%) therapy, 101 (87.8%) patients received statins treatment and seven (6.1%) patients were treated with intravenous thrombolysis (IVT) or mechanical thrombectomy.

**Table 1:** Baseline Characteristics of the VBD patients

Characteristics	Total (*n* = 115)	With IS Recurrence (*n* = 22)	Without IS Recurrence (*n* = 93)	*P* value
Age, y; mean ± SD	63.1 ± 10.7	64.9 ± 10.0	62.7 ± 10.8	0.389
Male (%)	88 (76.5)	16 (72.7)	72 (77.4)	0.641
Hypertension, n (%)	85 (73.9)	20 (90.9)	65 (69.9)	0.044
Diabetes mellitus, n (%)	38 (33.0)	12 (54.5)	26 (28.0)	0.017
IHD, n (%)	10 (8.7)	5 (22.7)	5 (5.4)	0.030
Hyperlipemia, n (%)	34 (29.6)	5 (22.7)	29 (31.2)	0.434
Smoke, n (%)	37 (32.2)	5 (22.7)	32 (34.4)	0.292
NIHSS, median (IQR)	3 (1–6)	4 (1–9)	3 (1–6)	0.194
Basilar artery geometry				
≥ 5.3 mm, 90th percentile	12 (10.4)	8 (36.4)	4 (4.3)	< 0.001
BA bifurcation, score = 3	7 (6.1)	4 (18.2)	3 (3.2)	0.032
BA laterality, score ≥ 2^a^	19 (16.7)	4 (18.2)	15 (16.3)	1.000
DID	15 (13.0)	8 (36.4)	7 (7.5)	0.001
ICAS	35 (30.4)	11 (50.0)	24 (25.8)	0.027
ECAS	15 (13.0)	3 (13.6)	12 (12.9)	1.000
In-hospital treatment				
Antiplatelet	98 (85.2)	18 (81.8)	80 (86.0)	0.869
Anticoagulant	23 (20.0)	2 (9.1)	21 (22.6)	0.236
Statins	101 (87.8)	20 (90.9)	81 (87.1)	0.897
IVT/thrombectomy	7 (6.1)	1 (4.5)	6 (6.5)	1.000

^a^One case of BA laterality could not be measured. Continuous variables are expressed as mean ± SD or median (IQR); other values are shown as n (%)

The mean diameters of the major intracranial arteries were measured as follows, BA 4.3 ± 1.1 mm, RVA 3.0 ± 1.0 mm, LVA 3.2 ± 1.0 mm, RICA 5.2 ± 1.0 mm, LICA 5.2 ± 0.9 mm, RMCA 2.7 ± 0.5 mm, LMCA 2.7 ± 0.5 mm. The median of BA diameter was 4.1 mm (IQR 3.6 mm - 4.8 mm), and the 90th percentile was 5.3 mm. 46 (40.0%) patients had basilar artery ectasia (BA diameter ≥ 4.6 mm), BA bifurcations of 73 (63.5%) patients were at the third ventricle floor (≥ grade 2), of these, seven (6.1%) were indented over the third ventricle floor, and BA laterality reached the lateral-to-lateral margin of clivus or dorsum sellae (grade 2) was seen in 19 (16.7%) patients. 15 (13.0%) patients had diffuse intracranial dolichoectasia (DID). Additionally, 35 (30.4%) patients had ICAS, nine (7.8%) had basilar artery atherosclerosis and 15 (13.0%) had ECAS.

During a 22 ± 6 months (ranged from two to 30 months) follow-up, 22 (19.1%) patients had IS recurrence, of these, 12 occurred in the posterior circulation, and ten in the anterior circulation. Four (3.5%) patients died, in detail, two died due to ischemic stroke recurrence, one for abdominal aneurysm rupture and one for lung cancer. Only one patient suffered non-fatal hemorrhage stroke. Univariate analysis **(**Table 1**)** identified hypertension (90.9% vs 69.9%, *P* = 0.044), diabetic mellitus (54.5% vs 28.0%, *P* = 0.017), and ischemic heart disease history (22.7% vs 5.4%, *P* = 0.030) as risk factors associated with IS recurrence.

Patients with BA diameter ≥ 5.3 mm (the 90th percentile) had more IS recurrence than patients with BA diameter < 5.3 mm **(**Table 1**)**, the percentage was 36.4% vs 4.3% (HR = 12.741; 95% CI, 3.376–47.878; *P* < 0.001). Other intracranial arterial geometry such as height of BA bifurcation (score = 3) (18.2% vs 3.2%; HR = 6.667; 95% CI, 1.373–32.372; *P* = 0.024), diffuse intracranial dolichoectasia (36.4% vs 7.5%; HR = 7.020; 95% CI, 2.199–22.418; *P* = 0.001), ICAS (50.0% vs 25.8%; HR = 2.875; 95% CI, 1.015–7.480; *P* = 0.027) was either associated with IS recurrence.

We introduced BA diameters into COX proportional-hazards model as a continuous variable, and found a significant relationship between BA diameter and IS recurrence, the crude HR per 1 mm-increase of BA diameter is 1.902 (95% CI, 1.439–2.514; *P* < 0.001). After adjusting for hypertension, ischemic heart disease, diabetes mellitus, height of BA bifurcation (score = 3), diffuse intracranial dolichoectasia, and ICAS, increasing BA diameter remain significantly associated with IS recurrence (HR = 1.756; 95% CI, 1.244–2.478; *P* = 0.001).

In multivariate analysis **(**Table 2**)**, BA diameter ≥ 5.3 mm (HR = 4.744; 95% CI, 1.718–13.097; *P* = 0.003) was an independent predictor of IS recurrence, along with diffuse intracranial dolichoectasia (HR = 3.603; 95% CI, 1.367–9.496; *P* = 0.010) and ischemic heart disease history (HR = 4.095; 95% CI, 1.221–13.740; *P* = 0.022).

**Table 2:** Multivariate Analysis of Predictors for Recurrence

Independent predictors	HR (95% CI)	*P* value
Ischemic heart disease history	4.095 (1.221–13.740)	0.022
Basilar artery diameter ≥ 5.3 mm	4.744 (1.718–13.097)	0.003
Diffuse intracranial dolichoectasia	3.603 (1.367–9.496)	0.010

Cox proportional hazards regression Forward LR method was used to examine the predictors of IS recurrence. The included covariates were conventional risk factors with a *P* value < 0.1 in the univariate analysis and MRI parameters such as basilar artery diameter ≥ 5.3 mm, the height of basilar artery bifurcation (score = 3), diffuse intracranial dolichoectasia, and intracranial atherosclerosis. All tests were 2-tailed, and *P* < 0.05 was considered significant

## Discussion

IS was the primary clinical manifestation of VBD; others included hemorrhage, SAH, compression of a cranial nerve, hydrocephalus et al. [16]. In this stroke registry-based study, 19.1% of VBD patients suffered a IS recurrence during a maximum 30-month follow-up. It seemed that stroke patients with VBD might have a higher risk of recurrence. For example, Flemming et al. selected 159 VBD cases from the radiological database which included not only stroke patients, after an average of 3.8-years follow-up, 44 patients occurred cerebral infarction or TIA. The 1, 5, and 10-year risk of an ischemic stroke was 6.1, 17.3, and 25.4% [2]. Passero et al. performed a clinical and imaging follow-up study and found that 75 (48%) of the 156 VBD patients had a stroke after an average of 11.7 years follow-up [1]. Besides, it seemed that the mortality (3.5%) in our study is lower than the previous study [17], this may be due to the relatively short follow-up time and exclusion of patients who died within 7 days because their diagnoses of stroke recurrence were mostly undetermined. Also, patients with no MRI were excluded; many of them were in unstable condition. These may lead to the underestimate of mortality. However, a previous study performed an average of 3.4 years follow-up of first ischemic stroke patients with IADE and also found a high stroke recurrence (58%) but low mortality (17%) [3].

**Fig. 2:**
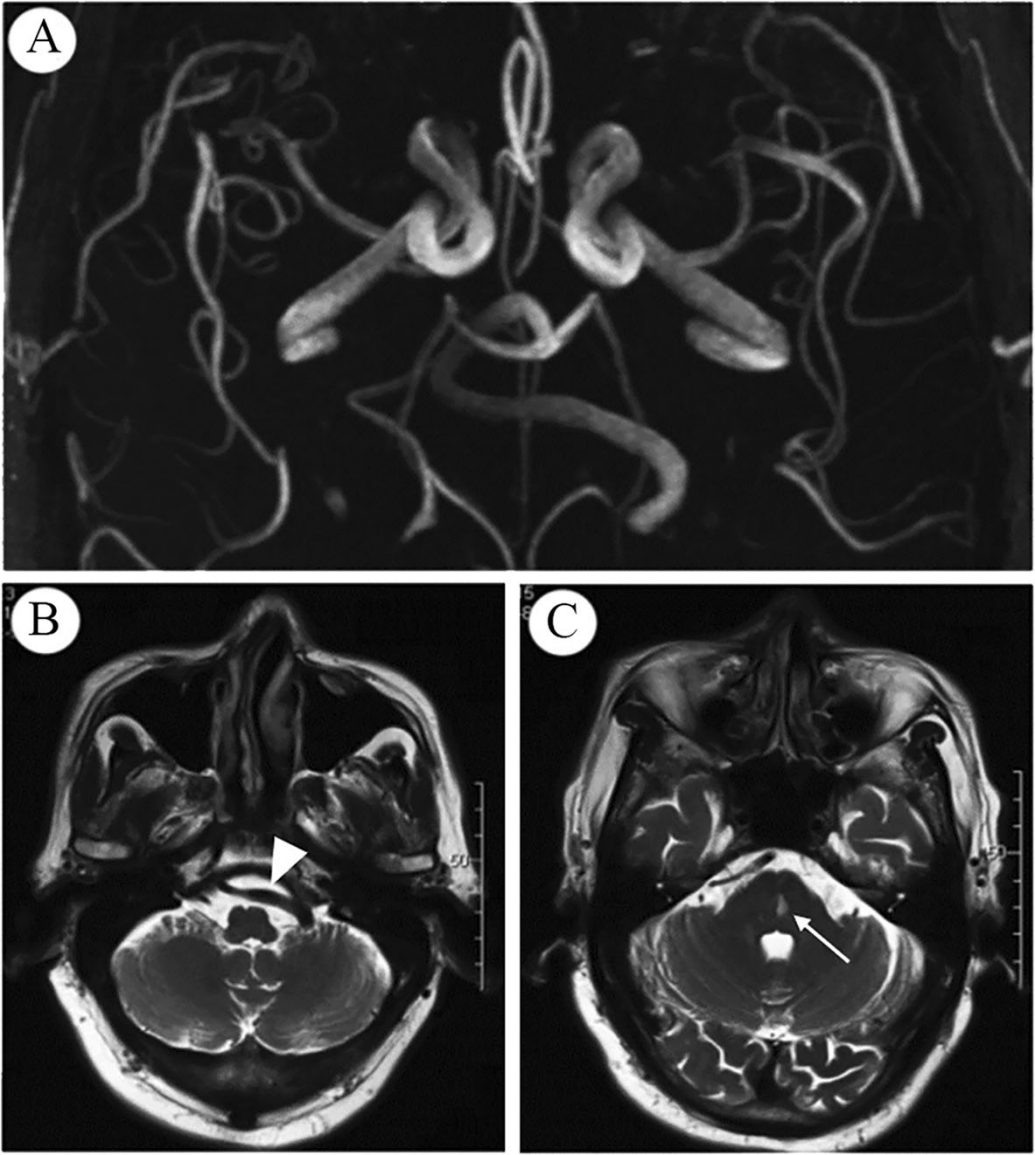
Brain ischemia due to Vertebrobasilar Dolichoectasia. A 76-year-old man admitted to the hospital with dizziness and slightly slurred speech. The 3D-TOF MRA showed extremely distorted basilar artery (A). The left vertebral artery (B) crossed the midline (white arrowhead) and merged the contralateral vertebral artery at the right cerebellopontine foot (C). In the contralateral side of the lateral displacement of the basilar artery tortuosity, a hyperintensity of lacuna lesions (C) can be seen in the center of the pons (white arrow), which may be caused by perforating artery occlusion due to the buckling or stretching of the circuitous basilar artery

Although VBD patients have a high stroke-recurrence rate, studies about the predictive factors of recurrence were limited. This study evaluated variables potentially associated with stroke recurrence in VBD patients and found that BA diameter ≥ 5.3 mm (90th percentile) was independently associated with IS recurrence. The higher IS recurrence among patients with extremely enlarged or kinking elongated arteries may be related to the multiple mechanisms that could lead to stroke occurrence [3], including local thrombosis, embolism, penetrating artery occlusion induced by compression or stretching of deep branches **(**Fig. 2**)** of the basilar artery. Interestingly, Pico et al. found that the BA diameter was also associated with a 5-year risk of death in stroke patients, the adjusted hazard ratio of stroke mortality was 1.23 (95% CI, 1.07–1.41) with per 1-mm increase in BA diameter [8].

We found another intracranial arterial geometry abnormality, the diffuse intracranial dolichoectasia, was correlated with IS recurrence. Patients with anterior circulation dolichoectasia have been reported in previous studies [1, 12]; however, the IS recurrence of patients with diffuse intracranial dolichoectasia was seldom mentioned [12]. Diffuse intracranial dolichoectasia patients suffered more IS recurrence mainly because it may be the severe form of arterial dolichoectasia. Brinjikji, W. et al. suggested that diffuse intracranial dolichoectasia is a systemic arteriopathy affecting multiple vascular beds, which may be different from a single intracranial artery dolichoectasia [12]. However, in this study, we could not uniquely say patients with diffuse dolichoectasia were different with the rest of patients with VBD alone, as we did not screen if any patients in this study have diseases that may often coexist with systemic vascular expansion, such as connective tissue disease, autosomal dominant polycystic kidney disease, or infection in this study.

This study found that previous ischemic heart disease was the independent predictors for IS recurrence in VBD patients. Interestingly, previous study found that IADE patients were eligible to have a previous myocardial infarction [18], and the coronary artery ectasia was also correlated with ischemic heart disease [19]. Concomitant coronary and basilar artery ectasia in stroke patients may suggest common pathogenesis [20]. However, in multivariable analysis, we did not find the statistical difference of vascular risk factors such as age, hypertension, diabetes mellitus, hyperlipidemia, smoking between the recurrence and non-recurrence groups. This may be due to the small number of recurrent patients in our study. Also, the negative findings of between-group differences refers to vascular risk factors might suggest the different pathogenesis between IADE and ICAS, as the characters above are widely accepted risk factors for atherosclerosis vascular disease.

In China, ICAS is the most common vascular lesions in patients suffered cerebral vascular disease, and the stroke recurrence is higher in patients with serve stenosis [21]. However, the relationship between large arterial atherosclerosis and dolichoectasia has always been debated. This study found that VBD patients had a frequent incidence of ICAS, and this proportion was higher in recurrent cases; however, we did not find its association with IS recurrence in multivariate analysis. IADE may differ with ICAS; autopsy study found that IADE was associated with rarefaction of elastic tissue of the tunica media with the degeneration of the internal elastic laminin [20, 22], while atherosclerosis always has a pathological change of plaque with a lipid core and the fibrous cap [23]. Also, hemodynamic abnormalities such as the wall shear stress, noticeable eddy currents in dolichoectasia vertebrobasilar artery were reported, and notably, without apparent arterial stenosis [24]. Interestingly, the ischemic lesions in the BA branches-supplied territories often exit at the contralateral side of the laterality of the BA [25], as is shown in Fig. 2. These findings suggested that the hemodynamic abnormality might promote the development of ischemic vascular events without atherosclerosis but due to stretching of the small branch vessels.

IADE and ICAS often coexisted in the large cranial arteries [1]. This implies that these two large artery abnormalities may share some common pathogenic factors. For example, matrix metalloproteinases (MMPs) are related to IADE as they could degrade various extracellular proteins including collagen, elastin, or proteoglycans that located in the tunica media [26]. Also, MMPs degrade the extracellular matrix and then causes vascular remodeling, finally leads to atherosclerosis [27]. And the high turbulent shear stresses and the region of flow separation and stagnation (especially in patients under long term hemodynamic changes such as hypertension) all give rise to thrombosis, causing occlusion of the perforating artery [23] or the distal embolism. However, it is also possible that IADE is merely a hint for severe atherosclerosis.

The widely accepted consensus of the secondary prevention for IS recurrence in VBD patients is lacking. The safety and effectiveness of antiplatelet or anticoagulant therapy in VBD patients has not yet been properly assessed [23]. Our study did not find the differences in treatment between groups referring to the IS recurrence, such as antiplatelet, anticoagulant, or statin. However, a small sample (13 patients) study favored anticoagulant to antiplatelet therapy because they observed a more favorable outcome [6], though another study cautioned against using either agents due to the risk of intracranial bleeding in VBD patients [28]. It seemed reasonable to give antithrombotic therapy conservatively in patients with a basilar artery diameter larger than 10 mm, in consideration of the high risk of rupture [23]. A favorable outcome was reported in patients with subarachnoid hemorrhage caused by posterior circulation fusiform aneurysms by using surgery or endovascular procedure [29]. However, randomized clinical trials are needed to assess the safety and efficacy of these approaches.

Our research has some limitations. First, this is a retrospective and hospital-based study, it was limited by case selection and referral bias, so the predictors of stroke recurrence in our study may not be an accurate reflection of the general population. Second, we did not include patients with suspected TIA or patients with negative CT/MRI findings, because it was difficult to give a definite diagnosis based on the retrospective chart review, as the mimic neurological deficits might be caused by epilepsy, peripheral vertigo, or syncope. Therefore, this may underestimate the true IS recurrence. Third, we did not use high-resolution MRI analyzing the vascular wall or the plaque stability as previous studies [30], as the negative arterial remodeling may underestimate arterial diameter [31]. However, this is a stroke registry-based study which patients were included prospectively and consecutively. The relatively large sample size may be powerful in analyzing the risk of IS recurrence in VBD patients. This study also pointed out concerns related to some specific VBD patients, such as those with combined anterior dolichoectasia, all of which may be helpful in guiding medical management of VBD patients, including improvement in patient counseling. Large prospective studies on this topic, including the use of high-resolution MRI or fluid dynamics needs to be expanded further.

## Conclusions

IS patients with VBD may suffer a high risk of recurrence. Larger basilar artery diameter or diffuse intracranial dolichoectasia may increase the risk of recurrence.

## Abbreviations

ACA: Anterior cerebral artery
BA: Basilar artery
CI: Confidence interval
CT: Computerized tomography
DID: Diffuse intracranial dolichoectasia
DSA: digital subtraction angiography
ECAS: Extracranial atherosclerosis
HR: Hazard ratio
ICA: Internal carotid artery
ICAS: Intracranial atherosclerosis
IHD: Ischemic heart disease
IS: Ischemic stroke
IVT: Intravenous thrombolysis
MCA: Middle cerebral artery
MMPs: Matrix metalloproteinases
MRI: Magnetic resonance imaging
NIHSS: National Institute of Health Stroke Scale
TIA: Transient ischemic attack
VA: Vertebral artery
VBD: Vertebrobasilar Dolichoectasia
